# Hyoscine and the Mydriatic Alkaloids

**Published:** 1910-12

**Authors:** J. M. Fortescue-Brickdale

**Affiliations:** Assistant-Physician to the Bristol Royal Infirmary; Lecturer on Pharmacology in the University of Oxford.


### HYOSCINE AND THE MYDRIATIC ALKALOIDS.

BY

J. M. FORTESCUE-BRICKDALE, M.A., M.D. Oxon.,

*Assistant-Physician to the Bristol Royal Infirmary;*
*Lecturer on Pharmacology in the University of Oxford.*

THE physiological phenomenon of mydriasis may be produced by the action of a number of alkaloidal bodies, and is known to bear a certain relationship to their chemical structure. A dilatation of the pupil may theoretically be produced by a paralysis of the circular muscle fibres of the iris, or of the endings of the oculomotor nerve, or by the stimulation of the radial muscle fibres, or of the sympathetic nerve endings. The mydriatic alkaloids (of which atropine is the best known) act in the first-mentioned manner—that is, they paralyse the sphincter fibres and the oculomotor nerve endings. Whether atropine also stimulates the sympathetic nerve endings is uncertain; it seems probable that it does not. Cocaine, on the other hand, owes its mydriatic effect mainly to its stimulation of the dilator fibres, and moreover it is without effect on the ciliary muscle and the light reflex. For this reason it is not usually classed among the mydriatics, which are most of them derivatives of a base known as tropine.

Tropine being a basic substance, is capable of combining with acids to form what are known as esters—the series of bodies so produced being called tropeines. Tropine has a somewhat complicated chemical structure; it is formed of a double ring of carbons, and contains in the group common to both a single atom of nitrogen :—

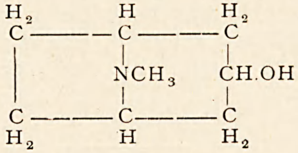

It also contains one hydroxyl (OH) group, and it is to this that the acids are attached, with the loss of the elements of water, when a tropeine is formed. Tropine itself has some action on the pupil, for it causes mydriasis in cats when given in toxic doses, but the tropeines show considerable diversity, some being actively mydriatic, and others being inert. Ladenberg, by an elaborate series of investigations, was able to show that in a large number of instances the mydriatic power of the tropeines depended on the chemical configuration of the acid "side chain," to which the basic tropine was united. Only those tropeines are mydriatic in which this acid contains a benzene ring, and an alcoholic hydroxyl (OH) group. Though Marshall, Jowett, and others have shown[1] that this law is not quite universally true, yet it obtains in a large number of cases. Thus the combination with tropic acid (atropine)

$$C_6H_5CH\begin{cases} CH_2OH \\ COOH \end{cases}$$

and that with mandelic acid (homatropine)

$$C_6H_5C\begin{cases} OH \\ CH_3 \\ COOH \end{cases}$$

are well-known mydriatics, whereas the lactic acid compound

$$CH_3CH,OH,COOH$$

[1] *Tr. Chem. Soc., Lond.*, 1900, 1906–7.

the cinnamic acid compound

$$C_6H_5CH : CH,COOH$$

are inactive, because in neither are both the required conditions present. A salicylic acid tropeine

$$C_6H_4OH,COOH$$

is also inactive, because the OH group is of the nature found in phenol, and not that of the ordinary aliphatic alcohol. This curious relationship has been found to apply to certain artificially produced bodies in which the base is not the same as tropeine. An example of this class is the substance known as euphthalmine.

Atropine, or tropyl-tropeine, is a crystalline body, only slightly soluble in water. It is optically inactive, its solutions failing to rotate the ray of polarised light either to the right or left. It will be observed that tropic acid

$$\begin{array}{c} H \\ | \\ C_6H_4—C—CH_2OH \\ | \\ COOH \end{array}$$

contains an asymetrical carbon atom, that is one of which all the four affinities are satisfied by different groups. It is therefore capable of existing in two stereo-isomeric forms, a right-handed and a left-handed variety. It is possible that it transmits this characteristic to its derivative, atropine—at any rate, dextro and lævo-rotary varieties of this substance are known, as well as the ordinary racemic or inactive body. Cushny[1] has shown that the alkaloid naturally occurring in belladonna is lævo-rotary, and is identical with the body known as hyoscyamine; in solution this changes into the racemic form, atropine. The dextro-rotary form does not occur naturally in the free state. The dextro and lævo-hyoscyamines have a somewhat different physiological action; with regard to the action on the central nervous system *d* hyoscyamine acts most powerfully, and *l* hyoscyamine least so, whereas the action of the latter variety on the iris and peripheral nerve endings is twice as strong as that of atropine, *d* hyoscyamine having hardly any action at all on these structures. Atropine, therefore, which

1 *J. Physiol*, 1904, xxx, 176.

contains equal moieties of both will act half as strongly on the peripheral nerve ending as l-hyoscyamine, though its central action is equal, owing to the presence of the dextro-rotary portion.

Pseudo-hyoscyamine, isolated from duboisia, is a crystalline alkaloid with a melting-point of 132–134°C. Large doses are said not to increase the pulse rate, and may decrease it. It is generally less toxic than atropine.[1] Cushny states that its mydriatic action is slight. Belladonnine and atropamine differ from atropine chemically and physiologically to a slight extent. Little is known about them.

The bodies known as daturine, duboisine, and mandragorine are mixtures of variable composition, and need not be further considered ; there remains, however, the interesting substance hyoscine, which is now somewhat largely used in medicine as a sedative and hypnotic.

The structural formula of hyoscine is not precisely known. Its empirical formula differs from that of atropine in containing one atom more of oxygen and two atoms less of hydrogen, being thus its oxidation product.[2]

Bender in 1892, and E. Schmidt and O. Hesse in 1893, came to the conclusion that hyoscine was identical with scopolamine, an alkaloid obtained from the root of scopola carniolica, a plant indigenous in parts of Austria, and official in the United States Pharmacopæia as a substitute for belladonna. In 1894 Hesse suggested that scopolamine was composed of two isomeric bases, one of which he called hyoscine and the other atroscine. The latter is optically inactive, the former, which is the natural base, is lævo-rotary.[3] Hyoscine in alcoholic solution containing a trace of alkali is according to Hesse quickly converted into the racemic form. Boiling with baryta water is said to split up scopolamine into tropic acid and a base scopoline (which has also been called oscine) $C_8H_{13}NO$, the melting-point of which is said to be 110° C (E. Schmidt). This base, it will be observed, contains two atoms of hydrogen less than tropine.

1 Buonarotti, *Arch. Ital. de Biol.*, xxiii. 1 and 2, 211, 1895.

2 J. Schmidt, *Pflanzen alkaloide*, 1900, p. 38.

3 Naylor, *Pharm. J.*, 1905, ii. 126.

The analogy of these bodies, the optically active and inactive hyoscines, to the optically active and inactive hyoscyamines is very striking. Lævo-rotary hyoscine has a powerful peripheral action, dextro-rotary hyoscine is almost inert. Both act similarly on the central nervous system.[1] The natural alkaloid is very similar to atropine in its peripheral action, but acts more powerfully and rapidly, though the effect passes off sooner.[2] In its cerebral action it differs from atropine. The stage of excitement is short or absent altogether, and soon a condition of marked depression and drowsiness supervenes. The excitability of the motor areas of the cortex to electricity is diminished, instead of being increased, as is the case with atropine in small doses. The temperature, which is raised by large doses of atropine, is unaffected by hyoscine. Occasionally symptoms of excitement have been observed, but these have been by some attributed to the presence of an impurity—apoatropine. According to Kessel,[3] this body is easily detected by adding a drop of potassium permanganate solution to the solution to be tested, which produces a brownish-yellow colour, if as little as one part in 20,000 of apoatropine is present. Scopolamine and atropine produce no such colouration.

The natural alkaloid is a thick syrupy liquid, and therefore unsuitable for therapeutic purposes. The salt most commonly employed is known as hyoscine or scopolamine hydrobromide, $C_{17}H_{21}NO_4HBr.3H_2O$, and is a mixture of the hydrobromides of the lævo-rotary and inactive isomers. It consists of colourless odourless crystals, with a bitter taste, soluble in two parts of water, thirteen parts of alcohol, and slightly soluble in chloroform and ether.[4] As a mydriatic a solution of 1 in 250 may be used; hypodermically the dose is 0.32 to 0.65 mgm. ($\frac{1}{200}$ to $\frac{1}{100}$ grain), which may be increased to 1.2 mgm. ($\frac{1}{50}$ grain). It may also be given by the mouth, the bitter taste being covered by chloroform water. The hydrochloride is said to be less easy to prepare in a state of purity.[5]

[1] Cushny and Peebles, *J. Physiol.*, 1906, xxxii. 501.
[2] W. E. Dixon, *Pharmacology*, 1906, p. 81.
[3] *Arch. Internat. de Pharm. et de Thérap.*, 1906, xvi. 1.
[4] *B. P. Codex*, 1907.
[5] Rüplmaar, *Wein. Med. Wchnschr.*, 1894, lxiv. 20.

The action of hyoscine varies in intensity in different individuals. Meijer[1] recommends that slightly smaller doses should be given to women than to men, and that increases in dosage should be gradual and cautious ; a failing heart and marked arterial degeneration are contra-indications for its use. Cushny points out that its comparative safety as a drug lies in the fact that it is easily and rapidly excreted or destroyed in the tissues.[2]

The following cases of poisoning by hyoscine or scopolamine have been recorded :—

(1) Worrall[3] in 1889 recorded a case in which $\frac{1}{100}$ grain (0.65 mgm.) caused faintness, a weak, rapid almost imperceptible pulse, widely-dilated pupils, and a cold, clammy skin. Recovery took place after ten hours, during which four injections of pilocarpine gr. $\frac{1}{10}$ were given.

(2) Gray[4] in 1892 observed two cases which illustrate the great variation in the effect of hyoscine on different individuals. In the first case, one of melancholia with marked motor unrest, gr. $\frac{1}{33}$ (2 mgm.) was necessary on most nights in order to obtain sleep. In the second, a healthy young woman suffering from tonsillitis was ordered gr. $\frac{1}{43}$ (1.5 mgm.) as a hypnotic. It immediately produced numbness of the body and limbs, dryness of the throat, dimness of vision, and rambling talk. In an hour she took a second dose, apparently of the same size, and became actively delirious for three hours, with a small, rapid pulse (92), fixed, dilated pupils, sighing respiration, and much general depression of function. The face was not flushed, and there was no laughter or noisy exhilaration. The case was treated by the injection of gr. $\frac{1}{4}$ morphine, and was almost well next day, though vision was not normal for three days.

(3) Pooley[5] in 1895 noted two cases in adult women, and one in a girl of 13, in which six instillations of a 0.2% solution of hyoscine hydrobromide into the conjunctival sac produced a

[1] Schmidt's *Jahrbüch*, 1900, 269, 222.
[2] *Pharmacology*, 1906, p. 290.
[3] Dixon Mann, *Forensic Med. and Toxicology*, 1908, p. 623.
[4] *Brit. M. J.*, 1892, i. 705.
[5] *Canadian Lancet*, Jan., 1895.

staggering gait, dry throat, rapid, irregular pulse (120), a sensation of "pins and needles" in the soles of the feet, and spasmodic contractions of certain facial muscles.

(4) Sharp[1] in the same year experimented on himself, and found that gr. $\frac{1}{100}$ (0.65 mgm.) instilled into the eye, or gr. $\frac{1}{33}$ (2 mgm.) *per os* produced first quickening and then slowing of the pulse, which was full and bounding in character, throbbing of the temples, dryness of the mouth, blurring of vision with dilated pupils, and staggering gait. His face became flushed, and there was mental confusion and somnolence. The symptoms passed off in twelve hours.

(5) Given[2] in 1904 recorded a case occurring in a man aged 69, who suffered from senile tremor and cramps, and who had previously taken the drug with benefit. Owing to a dispenser's error, the solution was made up with grams instead of grains, so that the patient received 4.8 mgm. or $\frac{3}{40}$ gr. (nearly $\frac{1}{10}$) in a single dose at bedtime. His throat became dry, and his speech thick almost immediately, and in half an hour he was deeply comatose, with stertorous breathing and dilated pupils. The face was flushed, the conjunctival reflex practically absent, and the pulse regular (80). The stomach was washed out, and hot coffee run in through the tube, and strychnine, morphine and caffeine were injected subcutaneously. He recovered in about seven hours.

(6) Morton's[3] case in 1896 was one in which gr. $\frac{1}{25}$ (2.6 mgm.) was instilled into the eye. In five minutes there was giddiness, dry mouth, staggering gait, and mental confusion. The patient gradually became unconscious, and there was complete muscular relaxation, flushed face, slow breathing, and full regular pulse. This condition lasted for four hours, and was succeeded by a cheerful delirium, in which the patient, though at times irritable, laughed a good deal and made jokes. After two hours she fell asleep. Recovery was complete with amnesia.

(7) Nicholson[4] in the present year has described the case of a

1 *Brit. M. J.*, 1895, ii. 1547. 2 *Lancet*, 1904, i, 24.
3 *Brit. M. J.*, 1896, i. 336.
4 *Lancet*, 1910, ii. 884.

boy, aged five years, who swallowed four or five tablets, each containing $\frac{1}{100}$ gr. hyoscine hydrobromide. He was shortly afterwards seized with convulsions. His temperature rose to 100° ; the pulse was 124 and bounding ; the skin was hot, dry, and flushed ; the pupils were fixed and dilated, and the patient quite unconscious. Pilocarpine hydrobromide, gr. $\frac{1}{4}$, injected produced marked and rapid improvement, and by the next day the child was quite well.

For the last eight years a number of cases in Germany and elsewhere have been treated by what was known as Schneiderlin's method of narcosis. This method, which consists of injecting small doses of morphine and hyoscine (scopolamine), has been specially practised in obstetric cases. The doses usually given are 3 mgm. (gr. $\frac{1}{200}$) scopolamine hydrobromide and 10 mgm. (gr. $\frac{1}{6}$) morphine hydrochloride. The injections may be repeated at a few hours' interval three or four times, but in these subsequent injections the morphine is usually omitted. This induces a condition known as "twilight sleep," in which there is considerable drowsiness and insensitiveness to pain, and subsequent forgetfulness of the events occurring while the patient was under the influence of the drug. Roith collected 4,000 cases from the literature with eighteen deaths, fourteen of which he considered due to the anæsthetic (mortality 0.35 per cent.). In other cases there were cyanosis, rapid, irregular pulse, vomiting, motor unrest, delirium, and heightened reflex excitability. Minor disturbances were extreme thirst, dryness of the mouth, and suppression of sweating and salivary secretion. Moreover, it has been shown that the drug easily affects the infant through the placental circulation, and some 20 per cent. of the children are born with more or less severe respiratory depression. The method appears to have no advantage over ordinary chloroform anæsthesia.

